# Discovering Links Between Side Effects and Drugs Using a Diffusion Based Method

**DOI:** 10.1038/s41598-019-46939-6

**Published:** 2019-07-18

**Authors:** Mohan Timilsina, Meera Tandan, Mathieu d’Aquin, Haixuan Yang

**Affiliations:** 1 Data Science Institute, Insight Centre for Data Analytics, National University of Ireland Galway, Galway, Ireland; 2 Discipline of General Practice, School of Medicine, National University of Ireland Galway, Galway, Ireland; 3 School of Mathematics, Statistics and Applied Mathematics, National University of Ireland Galway, Galway, Ireland

**Keywords:** Computational science, Network topology

## Abstract

Identifying the unintended effects of drugs (side effects) is a very important issue in pharmacological studies. The laboratory verification of associations between drugs and side effects requires costly, time-intensive research. Thus, an approach to predicting drug side effects based on known side effects, using a computational model, is highly desirable. To provide such a model, we used openly available data resources to model drugs and side effects as a bipartite graph. The drug-drug network is constructed using the word2vec model where the edges between drugs represent the semantic similarity between them. We integrated the bipartite graph and the semantic similarity graph using a matrix factorization method and a diffusion based model. Our results show the effectiveness of this integration by computing weighted (i.e., ranked) predictions of initially unknown links between side effects and drugs.

## Introduction

Adverse drug reactions (ADRs) or side effects are described as unexpected responses to drugs beyond their anticipated therapeutic effects^1^. ADRs can degrade the quality of human lives and even cause death in severe cases^2^. Each year, drugs approved by the Food and Drug Administration (FDA: https://www.fda.gov) are recalled because of their side effects, particularly when side effects are unexpected but discovered to be a major threat^3^. This process of post-market drug retraction is expensive. Therefore, the ability to judge the potential side effects of drugs as early as possible is crucial during the drug development process. Laboratory methods to predicting or assessing potential ADRs in the earlier phase rely on biochemical and cellular assays^4^. From the molecular perspective, recent work^5–7^ showed that RNA plays an important role in the development of human complex diseases particularly for microRNA. This can be used to evaluate drug efficacy based on drug-microRNA association. The drug-microRNA association constitutes an important pharmacological target, which result in improved specificity and lowered incidence of side effects.

Numerous initiatives have been carried out to develop in silico methods to predicting ADRs using diffusion based techniques^8–11^. In the context of ADRs and drug link prediction, computational studies use various databases including gene expression, pathways and chemical properties of drugs^11,12^. These computational approaches have been created by exploiting molecular features of chemicals and proteins, and phenotypic effects of drug treatments. Data mining techniques over web search logs have also been considered to study ADRs and drug safety surveillance^13^. It is very important to understand drug-drug interactions to study ADRs and Drug associations. To achieve this, *in vitro* experiments and clinical trials can be performed^14^, but systematic combinatorial screening of drug-drug interaction candidates remains challenging and expensive^15^. Researchers have thus attempted to collect drug-drug interactions from scientific literature and electronic medical records^16^ which can be very important in the absence of real *in vitro* data. It is also suggested that Natural Language Processing (NLP) has much potential for drug and side effect studies through the scientific literature. In biomedical text mining, named entities (genes, proteins, drugs, etc.) are recognized and the relations among them are extracted. Additionally, biomedical ontologies are utilized as sources of semantic information. Many NLP based models utilize such information to create word embeddings for semantic similarity measure^17^. Semantic similarities, which is core to several techniques used in health informatics and medical information retrieval^18^, have already provided quality results and showed how well they correlate with real human-judged similarity^19^. Motivated by this, we focus on the problem of ADR and drug link prediction by (i) creating a semantic similarity network of drugs using textual embedding methods, (ii) learning the diffusion weights of side effects in a side effect-drug network by matrix factorization, and (iii) diffusing the learned weights using a diffusion-based method.

The rest of this article is organized as follows. In the next section, we give a brief literature review on various related works. We describe our methodology and data sets in Section 3. In Section 4, we demonstrate the experimental results which validate the effectiveness of our approach. Finally, we draw conclusions and discuss our future work in Section 5.

## Related Work

In a biological context, diffusion-based approaches for predicting relations between diseases and genes are well studied. Network propagation has become a popular technique in computational system biology with a focus on protein function prediction and disease-gene prioritization^20^. Many methods that rely on biological information use protein-target as features. The assumption underlying these approaches is the idea that drugs with similar *in vitro* protein-binding profiles tend to exhibit similar side effects^21^. There are some methods that have been developed to determine the association between ADR and perturbed biological pathways because these pathways shared the proteins that the drugs target. Li *et al*.^22^ describe a chemical system biology approach to identifying the off-targets of drugs. However, these approaches are based on the accessibility of gene-expression data collected during the chemical perturbations produced by the drugs. The success of these methods depends upon the availability of 3D structures of the protein which limits their usability because of the higher cost involved.

Cowen *et al*.^23^ have claimed that network-based propagation is a powerful data transformation method of broad utility in biomedical research. There are different variants of network propagation proposed, such as random walk^24^ and PageRank search^25^ algorithms applied to a biological problem. Nitsch *et al*.^20^ showed that heat diffusion algorithms have the potential to help prioritizing disease gene associations and perform best among all network-based diffusion approaches.

Finding associations between side effects and a drug is a link prediction problem. Matrix factorization is widely used for link prediction, where the networks are represented as matrices having cells representing relationships. Therefore, according to Menon *et al*.^26^, link prediction can be treated as a problem of matrix completion. For example, low-rank matrix decomposition based on Singular Value Decomposition (SVD)^27^ has been used for this purpose. Another variant of matrix factorization called Non-negative Matrix Factorization (NMF)^28^ has also been used in link prediction tasks^29^. One of the advantages of using NMF-based matrix factorization is that it can easily integrate heterogeneous information^30^ and has non negative interpretable advantages. For multi-relational link prediction, tensor-based factorization is prominently used. The strength of tensors is that the multi-relational graph can be expressed in higher-order tensors which can be easily factorized. These models do not require a priori knowledge that needs to be inferred from the data, unlike graphical models such as Markov Logic Networks (MLN) or Bayesian Networks^31^. In recent studies, a node2vec^32^ approach was used to analyze different network neighborhoods to embed nodes based on the assumption of homophily as well as structural equivalence for link prediction in a homogeneous network for the same edge type. Due to high accuracy, the node embedding techniques^33^ are preferred but they also have some limitations. These methods actually require learning steps which might be unfeasible for large-scale networks which have millions of nodes^34^. Similarity-based propagation methods are also well studied in predicting the links in bipartite networks. The classic network based propagation in recommender system predicting most relevant objects for users^35^ predict the links between two dissimilar node types.

Our diffusion approach differs from the methods mentioned above in two important ways. First, those heat diffusion-based approaches described above are applied in a homogeneous network, where nodes and the edges are of the same type. While we consider heterogeneous networks and integrate them in an effective way. Second, we used 2 different networks, first for learning the seed nodes to carry side effect information in a drug-drug similarity network, and second to predict the associations between side effects and drugs. More specifically, we integrated NMF and heat diffusion methods to effectively handle the two different networks.

## Methodology

### Datasets

#### Construction of bipartite graph between side effects and drugs

The data sets we used are publicly available databases: (i) DrugBank (https://www.drugbank.ca) for drugs, (ii) SIDER (http://sideeffects.embl.de/) for drug side-effects, (iii) PubChem (https://pubchem.ncbi.nlm.nih.gov/) for compound IDs which are used to link drugs in DrugBank to the ones in SIDER. After the linking of drugs and side effects, there are 1020 unique Drugs and 5598 side effects. The edges between side effects and drugs are the facts reported in the SIDER database.

#### Construction of semantic graph between drugs

**Figure 1:**
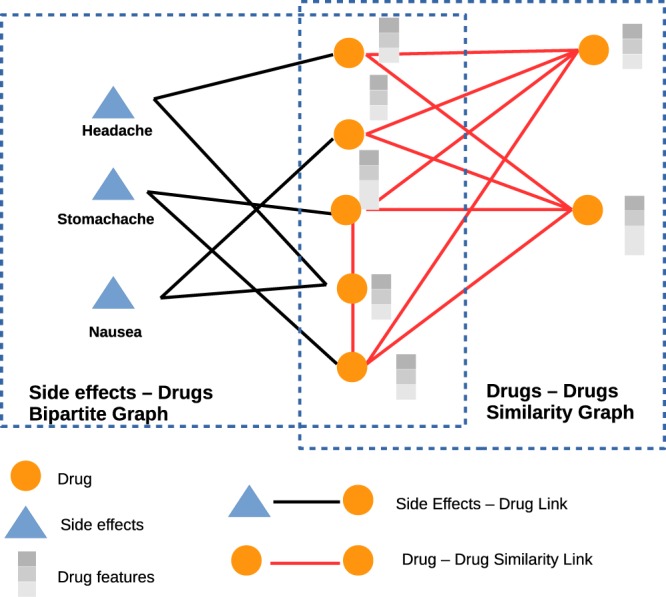
An example of side effect-drug bipartite graph and drug-drug similarity graph.

We constructed the semantic similarity between the drugs using a trained word2vec models. For this task, we used the open source skip-gram model provided by NLPLab (http://evexdb.org/pmresources/vec-space-models/wikipedia-pubmed-and-PMC-w2v.bin). This model is trained on all PubMed abstracts and PMC full texts (4.08 million distinct words) with 200 dimensions in combination with the latest Wikipedia dump. This model has also been used to extract chemical induced disease relations from the scientific literature^36^. The summary of the Network is shown in Table 1. The example of drug-drugs semantic similarity graph is shown in Fig. 1, the right side rectangle in the dashed line contains the drug-drug similarity graph and the left side rectangle in the dashed line contains the side effects-drugs bipartite graph.

**Table 1:** Summary of Side Effect-Drug and Drug-Drug Network.

Node Types	Property
Number of Drug Nodes	1020
Number of Side Effect Nodes	5598
Number of Side effect and Drug relationships	133750
Number of Drug-Drug relationships	519690

#### Heat diffusion model

Heat is a form of energy which can be transferred from a body with a high temperature to one with a lower temperature. Heat diffusion-based approaches have been successfully applied in various domains such as web spamming in web graph analysis^37^, recommender systems^38^ and disease-gene prioritization^20^. We model the diffusion of side effects in the drug network as a process of heat diffusion. In a drug-drug network, the drugs which are linked with side effect information act as heat sources, and have a very high amount of heat. These drugs initiate to influence other drugs and diffuse their influence on their neighbors. In this paper, we use heat diffusion models applied on an undirected graph where the links are weighted.

#### Diffusion on undirected weighted drug-drug semantic networks

A weighted graph is a graph where edges have weights. The edge weights correspond to the semantic similarity between the drug nodes. Consider a weighted graph representing a drug network such that *G* = (*V*, *E*, *W*) where V is the set of nodes, corresponding to drugs. In the case of an undirected and weighted graph, an edge (*v*_*i*_, *v*_*j*_) ∈ *E* is treated as a connection through which the heat flows and connecting nodes *v*_*i*_ and *v*_*j*_. A weight *w*_*ij*_ ∈ *W* is a *weight* score associated with the edge (*v*_*i*_, *v*_*j*_). The heat diffusion activation process is given by:

1 $${\bf{f}}(1)={e}^{\alpha {\bf{H}}}{\bf{f}}(0),$$

To model *H* or heat matrix for undirected weighted graph, we took the approach provided by Yang *et al*.^37^:

2 $${H}_{ij}=\{\begin{array}{ll}-(\frac{{\tau }_{i}}{{d}_{i}}){\sum }_{k:(i,k)\in E}{w}_{ik}, & {\rm{if}}\,j=i\\ \frac{{w}_{ji}}{{d}_{j}}, & ({v}_{j},vi)\in E,\\ 0, & {\rm{otherwise}},\end{array}$$

*τ* is the flag to check whether the node has any outgoing links. If there is any outgoing links then *τ* = 1 else *τ* = 0 and *d* is the degree of the node. The matrix *e*^*αH*^ is called diffusion kernel. The parameter *α* is called thermal conductivity. The higher the value of *α*, the faster the heat spreads in the network.

#### Computational complexity of heat diffusion models

For a large graph, the direct calculations of *e*^*αH*^ is time-consuming because the computation of the matrix exponential requires *O*(*N*^3^) operations^39^. We therefore adopted the discrete approximations proposed by Yang *et al*.^37^ which is $f(1)={(I+\frac{\alpha }{M}H)}^{M}f(0)$. *M* is a positive integer representing the number of iterations and *I* is the identity matrix. The parameter *α* is the heat diffusion coefficient. Specifically, after using the discrete formalization of the heat diffusion algorithm, the complexity reduces to *O*(*M*|*E*|*N*), where *N* is the number of nodes and |*E*| is the number of edges in the graph.

#### NMF-based matrix completion

The motivation for the matrix completion problem is to discover an unknown real matrix from a small subset of its entries. This problem comes up in many application areas and has received wide attention in the recommender system particularly after the Netflix (https://www.netflix.com) challenge to predict user ratings for movies. Taking inspiration from this idea, we made an initial prediction for unobserved values using the bipartite graph of side effects and drugs. We expect that there exists an ideal matrix that encodes the weights of relationships between all the side effects and drugs.

For a given bi-adjacency matrix $Y=[{y}_{ij}]\in {{\mathbb{R}}}^{mxn}$, where rows represent side effects, columns represent drugs, and non-zero elements represent known links, the goal is to complete this matrix for any side effect and drug pairs. In the matrix *Y*, each element *y*_*ij*_ (1 ≤ *i* ≤ *m*, 1 ≤ *j* ≤ *n*) belongs to boolean values of [0, 1]. Here *y*_*ij*_ = 0 means that no weight is provided by side effects *i* for drugs *j*, while *y*_*ij*_ = 1, is the diffusion weight given by side effects *i* for drugs *j*. Among different matrix completion algorithms, NMF is considered as the best for completing sparse matrix^40^ and performs best among other state of the art approaches such as Singular Value Decomposition (SVD)^41^.

To predict the initial weights, the NMF approach uses all the known weights to decompose the matrix *Y* into the product of two low-rank, latent feature matrices, one for the side effects, *S*_*m*×*r*_, and the other for drugs, *D*_*n*×*r*_, so that:

3 $$Y\approx \hat{Y}=S{D}^{T}=\mathop{\underbrace{[\begin{array}{l}{s}_{1}^{T}\\ {s}_{2}^{T}\\ \cdot \\ \cdot \\ \cdot \\ {s}_{m}^{T}\end{array}]}}\limits_{m\times r}\mathop{\underbrace{[\begin{array}{llll}{d}_{1} & {d}_{2} & \mathrm{...} & {d}_{3}\end{array}]}}\limits_{r\times n}$$

The latent feature vectors for side effects *s* and drugs *d* are *r* dimensional, where $r\ll min\{m,n\}$. The predicted weights for the side effect-drug pair (s, d) is given by $\hat{y}={s}^{T}d$. The NMF factorization problem in Equation 3 can be resolved by solving the optimization problem,

4 $$\mathop{{\rm{\min }}}\limits_{S\in {{\mathbb{R}}}^{m\times r},D\in {{\mathbb{R}}}^{r\times n}}\parallel (Y-S{D}^{T}){\parallel }_{F}^{2}\,{\rm{such}}\,\mathrm{that}\,\,S,D\ge 0$$

where *F* is the Frobenius norm.

#### Side effect propagation in a toy network

**Figure 2:**
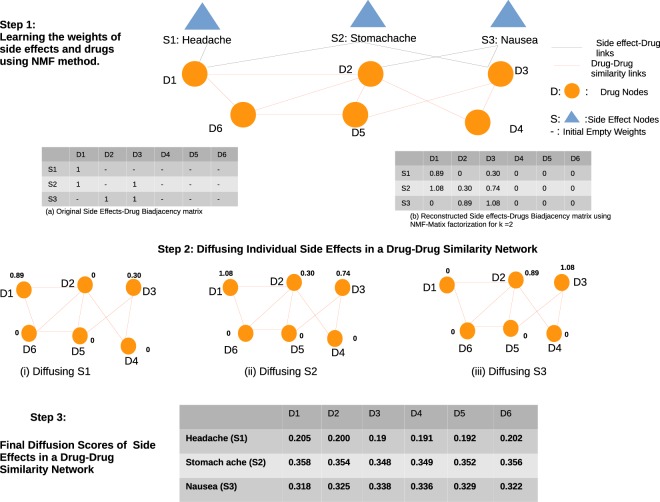
Side effects propagation in a drug-drug similarity network.

To illustrate our method, we show an example of propagating side effects in a toy network. In Fig. 2, step 1, there are two types of nodes and relationships. The nodes are Side effects and Drugs. The relationships between Side effects and Drugs are shown by black lines and the semantic relation between drugs is shown by red lines. The bipartite graph of Side effects and Drugs is shown as a biadjacency matrix (see Fig. 2 step 1(a)). The cells with 1 represent existing connections, and empty cells represent the absence of a known connection. To fill the empty cells we applied the NMF matrix factorization algorithm with *k* = 2. Once the matrix is filled, we obtain association scores of each side effects with the respective drugs as shown in the matrix in Fig. 2 step 1(b). These scores are now considered as heat weights for each side effect.

In step 2, we diffuse the weights of each side effect in the drug-drug similarity network. For instance, the initial heat weight vectors of side effect Headache is given by *f*(0) = [0.89, 0, 0.30, 0, 0, 0]. Now with this weight vector diffusing the side effects in a drug-drug similarity network with *α* = 1 and applying heat diffusion Eq. 1, the new diffusion scores of side effect Headache is given by f(1) = [0.20, 0.20, 0.19, 0.19, 0.19, 0.20]. From this computation we observe that the pair (Headache, Drug:D1) and (Headache, Drug:D6) are the first and second highest ranked pairs. In Fig. 2 at step 3 the drugs D2, D3, D4, D5 and D6 have new weights for side effect Headache which initially had no weights. This final diffusion score vector can be considered as the predicted impact of drugs on the corresponding side effect. With the same process, the other side effects Stomach ache and Nausea are calculated.

## Results and Evaluations

### Computation of heat diffusion scores of side effects in a drug-drug similarity graph

To fill the missing weights between side effects and drugs, we first applied the NMF algorithm. To choose the optimal number of latent factors in NMF we used a cross-validation method in the training sets. Once the weights are learned, we use these weights as the initial temperature for the heat diffusion process and diffuse them in the Drug-Drug similarity graph. There are two other important parameters: *α* which is the diffusion rate, and *M* the number of iterations. The parameter *α*, also known as thermal conductivity, plays an important role in the heat diffusion process. If *α* is set too high, heat diffuses too fast. Yang *et al*.^37^ found that in practical setting *α* = 1 and *M* = 30 is optimal in most cases.

### Evaluation

To evaluate the prediction ability of the NMF based Heat diffusion method we conducted a cross-validation by partitioning all side effects into ten folds and removing the links between side effects and drugs in the test set. We computed the heat diffusion scores and ranked all the side effects, and evaluated them using the area under the Precision-Recall curve (AUPR) score. The AUPR score is a standard performance metric which is commonly used in the machine learning community for quantifying the accuracy of link prediction algorithms^42^. This metric is considered more informative and robust in the presence of heavy class imbalance, as often the case in link prediction^43^.

**Figure 3:**
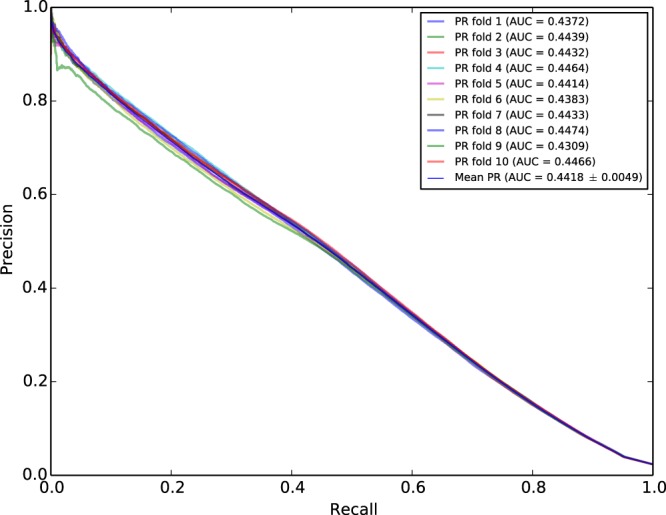
AUPR curve based on 10-fold cross-validation. The Blue line represents the Mean AUPR score.

Figure 3 shows the AUPR curve, with recall as the x axis and precision as the y axis, resulting from the 10 fold-cross validation of the algorithm. Each fold is represented by a color in the plot. The AUPR score for every fold is shown in the legend of the Figure. The standard deviation obtained from the 10 fold cross-validation is very small, with every curve showing very close to the others. From the Figure, we observe that the slope is facing downward from left to right, enforcing the notion that as recall increases, precision decreases.

#### Comparison with baseline link prediction algorithms

We evaluate our results from heat diffusion algorithms with baseline link prediction algorithms. For this, we implemented several algorithms for link prediction such as scores based on similarity metrics namely common neighbors, jaccard similarity, Adamic/Adar and resource allocation. These algorithms are also called node-based topological similarity algorithms because they can be viewed as computing a measure of “proximity” or “similarity” between nodes^44^.

We also compared the results from the heat diffusion algorithm with two widely used path based similarity algorithms called Katz and Personalized PageRank algorithm.

- **Random Baseline** This simply assign each candidate edge a random score.
- **Node Based** The link prediction metrics assign scores for each candidate edge. These metrics presented by/’^44^ are widely used in link prediction problem:
  1. Common Neighbors: $$score(x,y)=|{N}_{out}(x)\cap {N}_{out,in}^{^{\prime} }(y)|$$
  2. Jaccard’s Coefficient: $$score(x,y)=\frac{{N}_{out}(x)\cap {N}_{out,in}^{^{\prime} }(y)|}{{N}_{out}(x)\cup {N}_{out,in}^{^{\prime} }(y)|}$$
  3. Adamic/Adar: $$score(x,y)={\sum }_{z\in {N}_{out}(x)\cap {N}_{out,in}^{^{\prime} }(y)}\frac{1}{log|{N}_{out}z|}$$
  4. Resource Allocation: $$score(x,y)={\sum }_{z\in {N}_{out}(x)\cap {N}_{out,in}^{^{\prime} }(y)}\frac{1}{|{N}_{out}z|}$$
- **Path Based** Path based link prediction relies on the paths from one node to another. The two nodes are more likely to be connected the more paths there exist between them. We employed the following metrics to compute the score between two nodes:
  1. Katz: $$score(x,y)={\sum }_{i=1}^{\infty }{\beta }^{l}\cdot |path{s}_{x,y}^{ < l > }|$$
  2. Personalized PageRank: *score*(*x*, *y*) is explained as the probability of node *y* being present in a random walk that returns to node x with a probability *α* at each step, moving to a random neighbor with probability 1 − *α*

**Figure 4:**
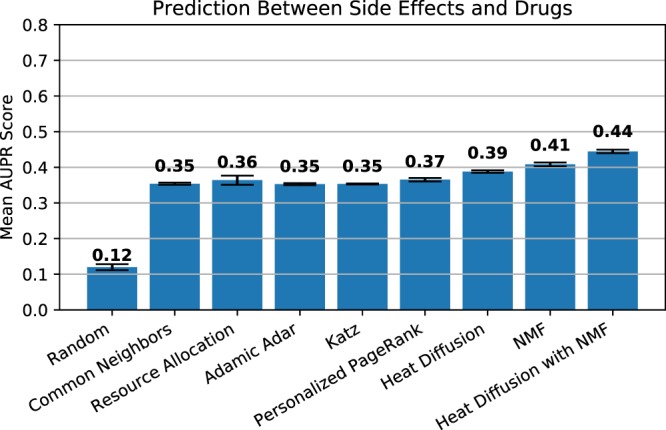
Link prediction results for the different state of the art methods. Each bar-chart shows the mean AUPR score for predicting links between side effects and drugs. The error bar represents the standard deviation obtained from the 10 Fold cross validation.

The results of our comparison with the baseline methods for link prediction are shown in Fig. 4. We observed that our approach of combining NMF with heat diffusion outperforms other state of the art link prediction methods. We performed the t-test to find out if there is a significant difference in the 10 fold cross validation results between our NMF-based heat diffusion method with other graph-based link prediction methods using a significance (*α*) level of 0.05. In the 10-fold cross-validated paired t-test procedure, we divide the test set into 10 parts of equal size. Each of these parts is then used for testing while the remaining 9 parts which are joined together are used as training sets. For each 10-fold cross-validation iteration, we compute the difference in performance between NMF based heat diffusion and baseline link prediction algorithms. Now, by making the assumption that these 10 differences were independently drawn and follow an approximately normal distribution, we can compute the t-statistic with 9 degrees of freedom according to Student’s t-test, under the null hypothesis that the NMF based heat diffusion and baseline link prediction algorithm have equal performance. After the t-statistic is computed, we can compute the p-value and compare it to our chosen significance level (*α*) of 0.05. If the p-value is smaller than *α*, we reject the null hypothesis. The p-values of the test are reported in Table 2.

**Table 2:** p-values of the t-test at significance level *α* = 0.05, *** indicates high significance.

Methods	P-value
Heat Diffusion With NMF Vs NMF	7.247e-12***
Heat Diffusion With NMF Vs Heat Diffusion	1.253e-15***
Heat Diffusion With NMF Vs Personalized Page Rank	2.2e-16***
Heat Diffusion With NMF Vs Katz	1.685e-14***
Heat Diffusion With NMF Vs Adamic Adar	2.2e-16***
Heat Diffusion With NMF Vs Resource Allocation	5.925e-10***
Heat Diffusion With NMF Vs Common Neighbors	2.2e-16***
Heat Diffusion With NMF Vs Random	2.2e-16***

We found that there is a significant difference between the prediction performed by NMF-based heat diffusion with the other state of the art methods. Although the NMF-based heat diffusion method outperforms the NMF methods only marginally, the p-value from the t-test (7.247e-12) suggests that there is a significant difference in AU-PR scores between these two methods. Similarly, NMF-based heat diffusion outperforms heat diffusion by 11% and there is a significant difference (p-value = 1.253e-15) in prediction performance between these methods.

#### Statistical significance of the side effects-drugs link prediction

**Figure 5:**
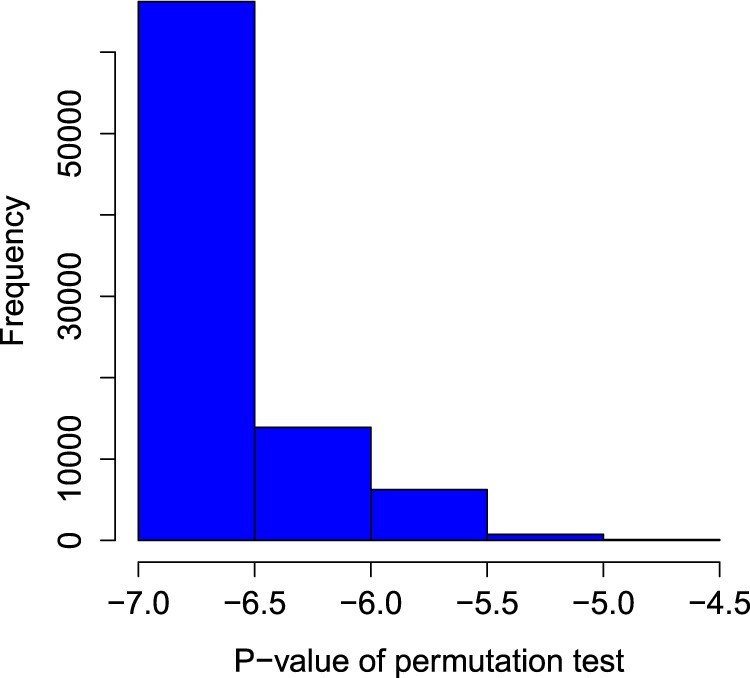
Histogram of P-values from Permutation test. The X-axis is in log base 10 scale.

To check the algorithmic significance of link prediction scores, we performed a permutation test for predicted side effects and drugs. We split the data randomly into training (75%) and testing (25%) sets. We recorded the NMF-based heat diffusion scores in a test set. After that, we randomize the drug-drug similarity graph by preserving the degree distribution and perform the diffusion in a random graph. The graph randomization process is repeated for 1000 times and p-values of every side effect and drug prediction score are computed as follows:

5 $$p-value(sid{e}_{-}effects,drugs)=\frac{{\rm{\Omega }}}{N}$$

where Ω is the number of randomly produced side effect-drug links which obtains higher heat scores than its actual predicted one. *N* is the total number of times the test is performed. The side effects and drug pair receiving higher p-values will be less likely to be an actual link because this pair will have a strong association with many randomly produced heat scores. The histogram of the p-values of our tests is shown in Fig. 5. We observed from the histogram that the large proportion of side effects and drug links are statistically significant (p-values are near to zero) and a very small amount of links have p-values > 0.05.

#### Performance of the NMF-based heat diffusion method using different training sets

**Figure 6:**
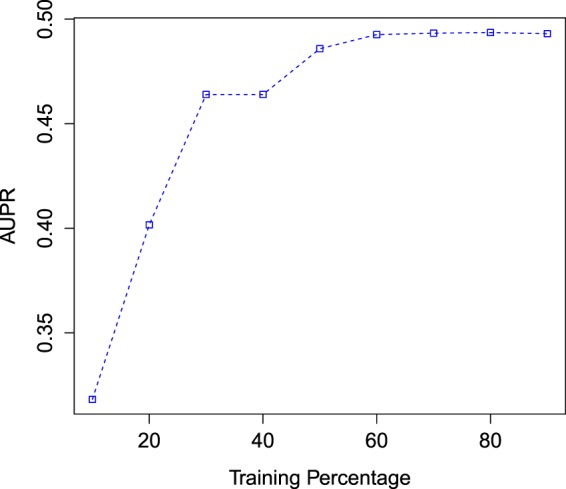
Performance of the NMF-based Heat diffusion algorithm using different training sizes. The Y-axis represents the mean AUPR score.

To test the robustness of the NMF based heat diffusion link prediction method, we used a different set of training data. From Fig. 6 we saw that when training size increased from 10% to 30%, there is a sharp increase in AUPR score. One of the reasons for the model performing badly when using a training set of 10% may be that the NMF method did not have sufficient information to learn the weights. Using a 50% training sample there is an visible improvement of the AUPR score. Using training sets larger than 50% does not appear to improve the results and the curve flattens out.

#### Examples of side effects prediction results

For illustration purposes, we randomly picked ten common and ten severe side effects^45,46^ which are shown in Table 3. We deleted the 80% of the known relationships of these side effects and let the NMF-based heat diffusion method predict those links.

**Table 3:** Examples of ranking performance for common and severe side effects.

Common Side Effects	AUPR	Severe Side Effects	AUPR
Constipation	0.89	Suicide	0.46
Diarrhoea	0.95	Depression	0.63
Nausea	0.95	Angioedema	0.72
Fatigue	0.92	Anaemia	0.82
Vomiting	0.97	Erectile dysfunction	0.66
Rash	0.96	Mania	0.54
Dizziness	0.96	Asthma	0.68
Insomnia	0.91	Gastric ulcer	0.33
Tremor	0.82	Muscle twitching	0.60

Most common side effects of the drugs are digestive related because many drugs are taken orally. Our approach showed good results for predicting vomiting, dizziness, diarrhea, and nausea. One of the reasons for that might be due to the same chemical components of the drugs triggering similar side effects. Very frequent side-effects, such as “vomiting” or “nausea” are found in SIDER databases, and they occur with many drugs, showing high AUPR scores. In the context of severe side effects, the algorithm performed poorly for gastric ulcers and suicide. These side effects are rare and serious, and might be caused by the use of multiple drugs or polypharmacy. The result of polypharmacy for a patient is a much higher risk of side effects, mostly because of drug-drug interactions. Our algorithm could not capture this effect sufficiently, leading to poor performance in the prediction.

## Discussion

Our results clearly show the combination of matrix factorization and heat diffusion-based technique outperform (i) node-based, (ii) path-based and (iii) NMF methods. In terms of AUPR results, heat diffusion with NMF leads to improvements of 6.81% on average over the NMF method and 11.36% over the heat diffusion method. Compared to path-based link prediction, heat diffusion has a marginal improvement over the personalized PageRank method. The important aspect of heat diffusion is that it represents an exponential sum which converges quicker, in most cases, than the geometric sum for path-based diffusion models like Personalized PageRank^47^. This can be advantageous in large graphs to get the desired results faster. In the biological context, similar work in prioritizing diseases and genes^20^ has already shown that heat diffusion-based ranking outperforms other diffusion methods for ranking disease-causing genes. Whereas, heat diffusion with NMF has produced accurate predictions with other node-based link prediction methods. Common Neighbors, Resource Allocation, Adamic Adar and Katz all have similar performances of predicting side effects with AUPR of around 0.35.

The main contribution of our method over the related state of the art is the combination of the following factors: (1) The presented method achieves better results through combining NMF with heat diffusion method; (2) The method does not use any laboratory data like other previous studies such as Drug-Drug interaction or other information from biomedical experiments, patient or report data. In this work, we took advantage of the NMF methods to make an initial prediction between side effects and drugs. In fact, we borrowed the concept of NMF based matrix factorization which has been successfully applied to missing value imputation from large and sparse matrices. The same concept has been applied to recommender system for predicting ratings for movies. To the best of our knowledge, it is the first time NMF has been incorporated with a heat diffusion method for side effect and drug link prediction. This concept relies on integrating two different graphs. We learned the weights for the drugs using side-effect and drug matrix factorization, from a side effect-drug bipartite graph. The learned weights are then propagated in the drug-drug semantic similarity graph. This graph is constructed from a word2vec pre-trained model using a Wikipedia- and PubMed-based corpus. By using the pre-trained model, the relationships between the drugs can be extracted, as distributed across millions of research articles and unstructured texts across the web. The semantic drug similarity network act as additional information and employing diffusion on this network improved the link prediction performance between side effects and drugs. From those results, we observed that the combination methods performed better than independent methods such as NMF or heat diffusion alone and also beats the other state of the art link prediction algorithms.

The most pressing future work stemming from the research presented here relates to automatically identifying the number of latent features for learning the initial weight of side effects and drugs from the bi-adjacency matrix without using cross-validation. In this study, we used all the scores between drug pairs given by the word2vec models. Taking all the semantic scores between the drugs may contain noise in the networks. It would be ideal to study the sensitivity of such similarity networks for side effect and drug link prediction. In this work, we have only used the drug-drug similarity network by using a pre-trained word2vec model. We believe integrating with other heterogeneous information networks would help exploiting complementary information and would improve prediction performance. The work by Chen *et al*.^48,49^ already demonstrated the efficacy of using heterogeneous information networks to predict miRNA - disease association and drug-target interaction by using a simple random walk model. In our future work, we consider integrating different heterogeneous information networks such as a drug-drug functional similarity network and a side effects similarity network, and implement our heat diffusion-based method to predict side effect and drug associations. In the current version, our model cannot predict the side effects of combinations of drugs, through drug-drug interactions. This is a very important issue in polypharmacy which remains a challenge with significant implications for patient mortality. There have also been some work^50^ using semi-supervise machine learning methods to predict drug combination.

## Conclusion

This paper presented a work on predicting links between side effects and drugs using a heterogeneous graph. To this end, a novel method incorporating matrix factorization and heat diffusion was applied for a side effect and drug association prediction task. The performance of the combined NMF and heat diffusion model was compared with other state-of-the-art methods, showing that the proposed method significantly outperforms the others. The limitation of our approach is in the construction of semantic similarity. In this work, we did not apply any semantic similarity threshold. This might have introduced some noise in the graph which might have influenced the prediction performance.

## Data Availability

The datasets generated during and/or analysed during the current study are publicly available in the Github repository https://github.com/timilsinamohan/sideeffects.
